# Exploring Red Wine Aging: Comparative Analysis of Cellar and Sea Underwater Aging on Chemical Composition and Quality

**DOI:** 10.3390/foods13121812

**Published:** 2024-06-08

**Authors:** Nicola Mercanti, Ylenia Pieracci, Monica Macaluso, Massimo Fedel, Francesco Brazzarola, Fabrizio Palla, Piero Giorgio Verdini, Angela Zinnai

**Affiliations:** 1 Department of Agriculture, Food and Environment, University of Pisa, Via del Borghetto 80, 56124 Pisa, Italy; nicola.mercanti@phd.unipi.it (N.M.); monica.macaluso@unipi.it (M.M.); angela.zinnai@unipi.it (A.Z.); 2 IFN CNR: Istituto di Fotonica e Nanotecnolgie, Consiglio Nazionale delle Ricerche, Via Trasea 7, 35131 Padova, Italy; massimo.fedel@pd.ifn.cnr.it; 3 Antares Vision.I., Via del Ferro 16, 25039 Travagliato, Italy; francesco.brazzarola@antaresvision.com; 4 INFN Pisa Section, Largo Bruno Pontecorvo 3, 56127 Pisa, Italy; fabrizio.palla@cern.ch; 5 European Organization for Nuclear Research Espl. des Particules 1, 1211 Meyrin, Switzerland; piero.giorgio.verdini@cern.ch; 6 Interdepartmental Research Centre “Nutraceuticals and Food for Health”, University of Pisa, Via del Borghetto 80, 56124 Pisa, Italy

**Keywords:** TDLAS, anthocyanins, phenols, refinement, thermodynamic analysis

## Abstract

The aging process of wine is influenced by various factors, including the presence of oxygen, the temperature, and the storage conditions. While oxygen can have both positive and negative effects on wine quality, temperature fluctuations during storage can impact its chemical composition. This study has investigated the aging of Merlot and Sangiovese wines under traditional cellar conditions and underwater, exploring the influence of storage parameters on their chemical evolution. Analyzing parameters such as temperature, pressure, and chemical composition, the research revealed subtle but significant changes in the wines over time. Both wines showed a gradual reduction in total phenols, anthocyanins, non-flavonoid compounds, and total sulfur dioxide, irrespective of the storage conditions. Preliminary findings suggested that aging wine underwater does not induce significant alterations in its fundamental characteristics compared to traditional cellar aging. These results contribute to a deeper understanding of wine aging processes and highlight the importance of storage conditions in preserving wine quality. Further research is needed to fully elucidate the complexities of underwater aging and its broader implications for wine production.

## 1. Introduction

Wine is a complex beverage whose evolution depends not only on its biophysical composition but also on the characteristics of the container in which it is placed. Among the main factors influencing the lifespan of wine, oxygen and temperature play crucial roles. The presence of oxygen in wine may have positive or negative effects depending on the stage of the production process [1]. It is beneficial for yeast multiplication during fermentation, for polyphenolic component polymerization, and for a reduction in odors [2]. Conversely, oxygen plays a detrimental role as it is able to oxidate aromatic compounds, ethanol, and phenols, particularly anthocyanins, imparting negative features [3,4]. Oxidative phenomena explain the color changes that the wine undergoes during maturation [2,5]. Indeed, anthocyanins, important phenolic compounds abundant in red young wines, tend to diminish significantly due to oxidative processes, copolymerization with other phenols, and polymerization of their non-oxidized and oxidized forms, resulting in a shift towards garnet red [6].

Oxygen, on the one hand, favors the growth of yeasts during fermentation and the polymerization of polyphenolic compounds, while on the other hand, it can lead to the oxidation of aromas and phenols, compromising the quality of the wine. This delicate balance between benefits and risks is fundamental to the production of high-quality wines and its role varies according to the stages of the production process.

Successful wine aging is related to the diffusive process and extraction of noble compounds from the wood and initial wine composition introduced into the container. Wines capable of sustaining wood aging should be transferred to barrels early (after malolactic fermentation), with a faster evolution in small barrels, characterized by a greater amount of dissolved oxygen per unit volume of liquid. However, the laws governing barrel aging are not yet fully understood, making proper product evolution somewhat random. Wines with high polyphenol content, especially tannins, respond more positively, while those poor in these components may be overly imbued with wood aromas and advanced oxidative characteristics [7]. The typical method for aging wine happens in the cellar storage, where the temperature typically ranges between 15 and 17 °C, providing the optimal conditions for maturation [8]. Exposure to light, particularly UV radiation, is able to trigger the formation of reactive oxidative species in wine. To mitigate this, minimal light exposure and the use of tinted glass bottles are recommended during storage [9]. Moreover, maintaining a relative humidity of around 70% is considered ideal as it helps to regulate the permeability of corks to oxygen, thereby preserving the quality of the wine. These specific storage parameters explain why many wine cellars are constructed underground [10]. In recent years, underwater ageing of wine has captured the attention of researchers, producers, and enthusiasts [8]. This innovative ageing method, inspired by the discovery of well-preserved bottles from marine wrecks, has fueled a growing curiosity about its effects on the chemistry and sensory profile of wines. Since it is not known how the maturation of wine underwater affects the chemical composition of the wine, differences between the same wines made from the same varieties aged in the cellar and in an underwater spring were investigated, in order to test the technological possibility to develop an underwater cellar that would not alter the characteristics of the wine. 

## 2. Materials and Methods

### 2.1. Sea-Aged Wine Protocol and Packaging

A protocol for wine underwater refinement was developed (Table 1). Bottles of Sangiovese 2019 and Merlot 2020, capped with a monoblock cork and shellac capsule and wrapped with a protective film, were stored both under the sea and in the cellar with an upright orientation and then analyzed for their chemical composition at fixed intervals. In detail, the batches of wine bottles to be refined under the sea were composed of 8 cages, each containing 1 bottle called “Sentinel”, which was a bottle of Sangiovese 2019 containing a sensor, and 15 bottles made up of 3 bottles from each lot. The bottles had been prepared and stored in a fridge at 4 °C for two days and then the samples to be refined under the sea were dipped underwater at a depth of 25 m below the sea level, while the control samples were placed in the cellar. The arrangement of the cages underwater was such that air current movements would be allowed without any obstructions. Both the samples refined under the sea and in the winery were periodically analyzed. Each sampling consisted of 3 bottles from each batch for a total of 21 bottles (15 from each cage and 6 from the cellar).

### 2.2. Sensors and Data Read Out

Sensors were specifically assembled to measure and record the pressure and temperature parameters of the wine bottles to achieve the objectives of the present work. The sensing element was a miniaturized MEMS high resolution altimeter sensor from TE Connectivity with an SPI and I2C bus interface. The sensor had a pressure accuracy of greater than 40 mbar and a temperature accuracy of 2.5 °C, with a resolution of 0.01 mbar and 0.002 °C. The sensor was interfaced to a micro-controller esp8266. Each assembly was powered with a lithium-based battery wrapped into a plastic bag and put inside the bottle.

### 2.3. Chemical Analyses

All chemical determinations necessary for the characterization of the wines (sugar content (hexoses g/L), titratable acidity (tartaric acid g/L), pH, L-malic acid (g/L) and potassium (mg/L)) were performed as previously described by Bianchi et al. [11]. The other determinations, including alcohol content (%*v*/*v*), AVN (net volatile acidity (g/L acetic acid)), dry extract (g/L) and ash (g/L), were carried out according to the OIV methods 2021, while the total polyphenols (g/L catechins), total anthocyanins (g/L malvin) and decolorable anthocyanins (g/L malvin) and total SO_2_ were measured as reported by Mercanti et al. [12].

### 2.4. Non-Destructive Analyses (EVO-1O_2_)

The laser spectroscopy-based technology EVO-1O_2_ was employed for bottle headspace atmospheric control. The instrument uses a laser beam of minimal intensity that is able to traverse the bottle headspace which might be undergoing some modifications that could be related to the headspace composition. Indeed, the transmitted laser signal shows adjustments in both wavelength and amplitude as a consequence of the interaction with the gaseous molecules, enabling the quantification of the oxygen content.

### 2.5. Statistical Analyses

One-way analysis of variance was performed using CoStat, Version 6.451, CoHort 6.0 Software (Pacific Grove, CA, USA) to assess the presence of significant differences on the compositional parameters among the investigated samples, while the JMP Pro 14.0. statistical package (SAS Institute; Cary, NC, USA) was used to evaluate the presence of significant differences in the relative content of the identified chemical classes of the volatile profiles. For both the analyses, the means were separated by Tukey’s post hoc test using a *p* ≤ 0.05. Each analysis was performed in triplicate.

## 3. Results

### 3.1. Preliminary Results

The aging of wine in glass bottles is influenced by numerous operating parameters that are able to affect the type of final product. In particular, the gaseous diffusion kinetics of the oxygen present in the bottle headspace is decisive for the evolution of the structural component, with particular reference to the phenolic compounds [13,14]. In the present experimentation, after analyzing the wine before the underwater refinement (Table 2), two different capping systems were evaluated for their ability to prevent oxygen entry.

- The cork, which represents the most used capping system in the wine sector.
- The crown cap, or screw cap, which is the most effective barrier against the entrance of oxygen from the atmosphere outside the bottle.

**Figure 1:**
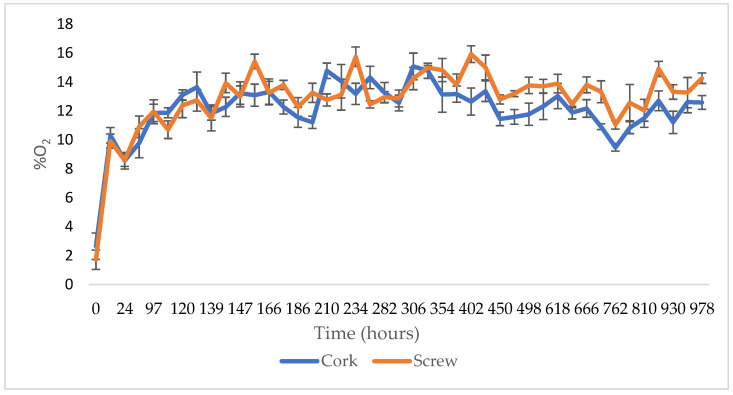
Evolution of oxygen concentration using different capping systems (cork and screw cap).

As evidenced in Figure 1, both the tested caps showed a similar oxygen permeability rate. Thus, for the experimentation the classic cork stopper was preferred as it is universally known and mostly employed in the wine sector. All the bottles capped with a cork had also been waxed in order to avoid the entrance of water during the refinement process.

### 3.2. Chemical Composition at the Beginning of Experimentation

At the beginning of the experiment, both wines (Merlot and Sangiovese) were chemically analyzed and the results are shown in Table 2.

The analysis of these initial parameters allowed us to verify the suitability of the wines for refinement in the bottle. Specifically, a good alcohol content and pH, a low sugar content (essential to avoid the start of undesired fermentation), a good level of titratable acidity (able to impart to the wine a good boost of acidity during refinement), and an adequate level of glycerin (necessary to keep the wine ‘soft’ during refinement in the bottle [15]) were features evidenced for the analyzed wine that indicate the suitability of the product for the refinement in the bottle, since they are required for avoiding depauperation of the product, especially under poorly studied conditions, like the undersea storage.

**Figure 2:**
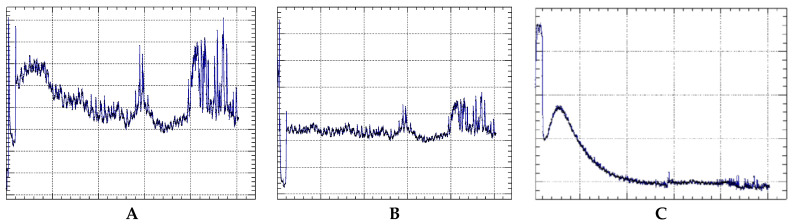
Trend of the pressure (**A**) and temperature (**B**) variables and evolution of the volume (**C**) of the headspace of the bottles during the entire aging period, as a function of time.

Figure 2 (a and b, respectively) shows the temperature and pressure trends in the bottles as functions of time. The graphs evidenced the correlation between the temperature and the pressure, as explained by Equation (1), in the first two days after the preparation, during which samples were stored in the fridge.

(1) $$\left(\frac{\frac{p}{1100\;mbar}}{{\left(\frac{T}{288\;K}\right)}^{6.5}}\right)=constant$$

where p is the pressure, T is the temperature, and the exponent 6.5 is the adiabatic lapse rate. In this equation, the ratio of the pressure to a reference pressure (1100 mbar) is divided by the ratio of the temperature to a reference temperature (288 K) raised to the power of 6.5. 

Afterward, during the underwater period, graphs evidenced a nearly constant temperature (with some day–night variations) for about three weeks, followed by some temperature peaks, corresponding to increased pressures, probably related to two particularly warm periods in August 2021. The behavior of the gas followed a polytropic law, a mathematical equation (Equation (2)) describing the relation between pressure and volume during a gas reversible process. It is expressed as follows:

P × Vn = constant(2)

In this equation, P represents the pressure, V the volume, and n the polytropic index. 

The polytropic law is commonly used to analyze processes in thermodynamics and fluid mechanics, such as compression or expansion of gases in turbines or compressors. By applying this law, it is possible to determine the mutual effects of changes in gas pressure and volume during these processes.

Following this constant, we applied the polytropic law to the system under consideration, according to Equation (2). Figure 2 clearly evidenced that although the temperature remained stable, the pressure dropped down from about 1200 mbar to 1100 mbar, particularly in the two weeks following the wine submersion. The decrease in the pressure although the constant temperature might probably be related to oxygen interactions with the wine, both of chemical and physical origins, due to the different oxygen dissolution in the wine before and after the water submersion. From our calculations, however, we expected only small variations in the dissolved oxygen rate in the wine in relation to the temperature variation, hence the main cause of the great dissolved oxygen levels might be related to chemical reactions. The increasing dissolved oxygen level in wine is also related to an increased degree of tannin polymerization [16]. Oxygen interacts with the phenolic components in the wine, including tannins, thus promoting the formation of bonds among them [17]. This process, known as tannin polymerization, contributes to the enhancement of the wine structure and complexity, influencing its organoleptic properties such as color, taste, and mouthfeel [18].

### 3.3. Chemical Analysis Wine

Understanding the evolution of wine compounds during storage is crucial for winemakers and oenologists to optimize aging processes, thus achieving desired quality profiles. By comprehending the influence of different storage conditions, such as the cellar or deep sea, on the chemical composition, winemakers can make informed decisions to enhance the quality and characteristics of the final product. The study aimed to elucidate the mechanisms involved in the maturation of wines under sea conditions and compare them with the traditional cellar aging. The chemical analyses carried out in the study offered a comprehensive view of the changes that occur in the composition of wine during ageing. Advanced analytical techniques, including sensors and laser spectroscopy, were employed to monitor the physical parameters that influence bottle aging, such as temperature, pressure, and headspace gas composition. Samples of Merlot and Sangiovese wines were collected at different stages of aging and subjected to comprehensive compositional analysis. This encompassed the evaluation of macro-parameters such as alcohol content, pH, titratable acidity, and residual sugars, as well as the assessment of structural and chromatic components, including total phenols, total and anthocyanins, and non-flavonoid compounds and the analysis of sulfur dioxide, an additive widely used in oenology due to its antiseptic, antimicrobial, antioxidant, and antioxidase properties that ensure the protection of wine.

#### 3.3.1. Merlot Wine

**Figure 3:**
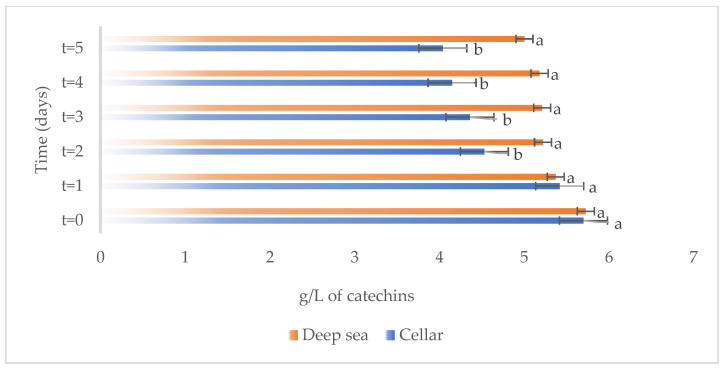
Total phenols present on the samples considered (Merlot both under the sea and in the cellar). Letters (a,b) indicate significant differences (*p* < 0.05) over time, after ANOVA analysis of variance.

**Figure 4:**
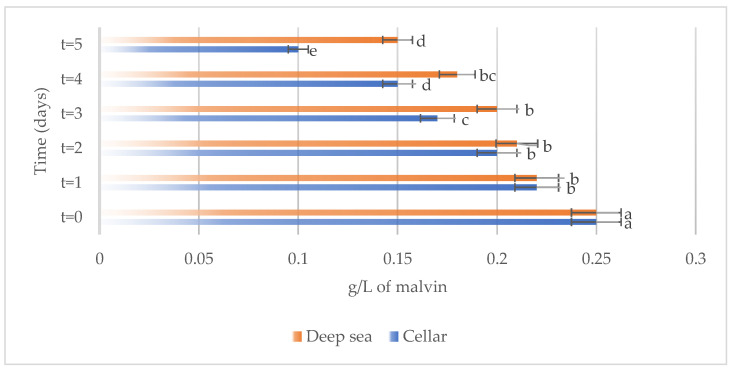
Total anthocyanins present on the samples considered (Merlot both under the sea and in the cellar). Letters (a–e) indicate significant differences (*p* < 0.05) over time, after ANOVA analysis of variance.

**Figure 5:**
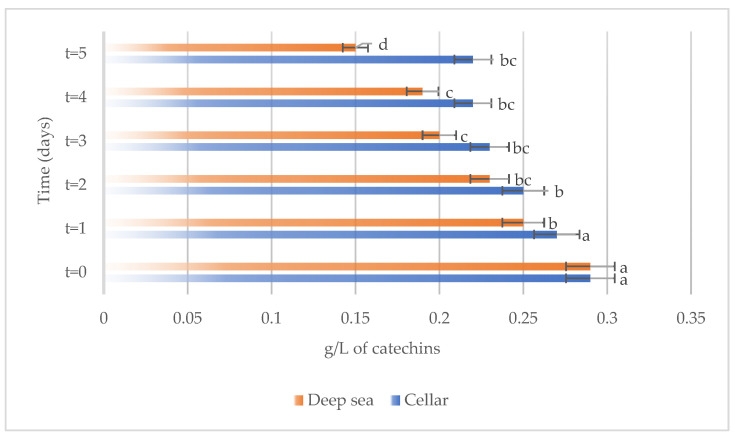
Non-flavonoid compounds present on the samples considered (Merlot both under the sea and in the cellar). Letters (a–d) indicate significant differences (*p* < 0.05) over time, after ANOVA analysis of variance.

**Figure 6:**
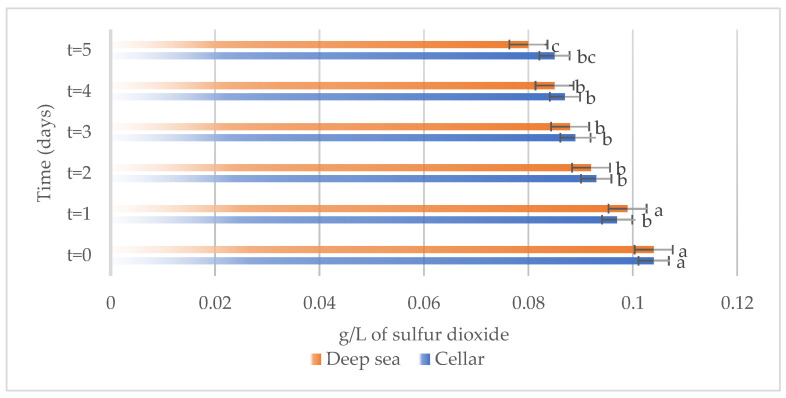
Total sulfur present on the samples considered (Merlot both under the sea and in the cellar). Letters (a–c) indicate significant differences (*p* < 0.05) over time, after ANOVA analysis of variance.

The variations in the chemical parameters, total phenols, total anthocyanins, non-flavonoid compounds, and total SO_2_ occurring in Merlot wines stored under different conditions, both in the cellar and underwater, are reported in Figure 3, Figure 4, Figure 5 and Figure 6, respectively.

Total phenols were found in similar amounts in the wines subjected to aging in both the investigated conditions. During the wine maturation, both wines aged in cellar and deep sea underwent a slight decrease, as evidenced in Figure 3. However, while total phenols did not show a significant reduction in wines aged in deep-sea conditions, whose concentration remained relatively stable over the storage period, they underwent a significant decrease in the in the cellar.

Regarding total anthocyanins (Figure 4), a decrease was noticeable over time in the wines stored both in the cellar and in deep-sea conditions, probably associated to a loss of pigmentation, as anthocyanins are molecules responsible for the red/purple colors [19]. However, wine aged in the deep sea seemed to be able to better preserve the anthocyanin content during the aging period when compared to the product refined in the cellar.

Non-flavonoid compounds (Figure 5), including various phenolic components of the wine, showed a slight decrease during the storage period in the wines maintained both in the cellar and under the sea. This decrease could be attributed to oxidation reactions or other chemical transformations that occur during the storage process. These results highlighted the dynamic nature of wine and its susceptibility to changes in composition during storage [20]. The variations observed in the wine composition emphasize the importance of proper storage conditions and the impact they may have on the wine chemical profile. Further studies and analysis, however, are necessary to fully understand the mechanisms regulating these changes and their implications on the overall quality and sensory characteristics of the wine.

Total sulfur dioxide, which can induce changes in the sensory characteristics of the wine [12], showed a consistent decrease during the storage period in the wines maintained in both the cellar and under the sea (Figure 6). This could be the result of oxidation processes able to reduce the presence of total sulfur in the wine [21].

#### 3.3.2. Sangiovese Wine

**Figure 7:**
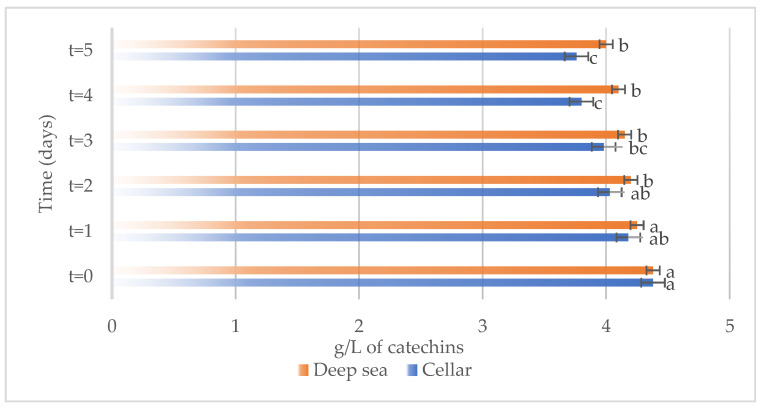
Total phenols present on the samples considered (Sangiovese both under the sea and in the cellar). Letters (a–c) indicate significant differences (*p* < 0.05) over time, after ANOVA analysis of variance.

**Figure 8:**
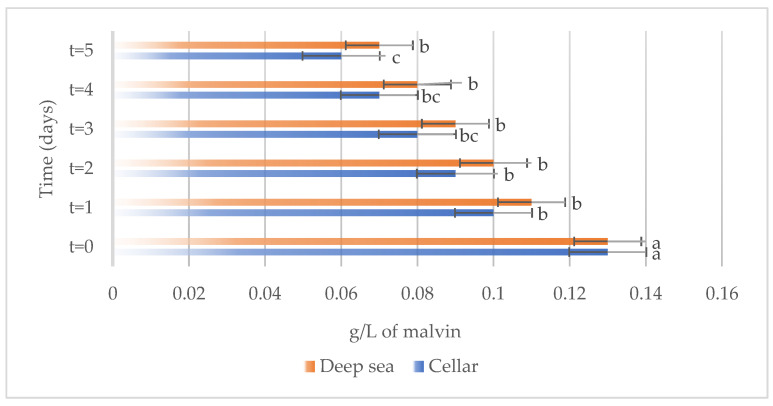
Total anthocyanins present on the samples considered (Sangiovese both under the sea and in the cellar). Letters (a–c) indicate significant differences (*p* < 0.05) over time, after ANOVA analysis of variance.

**Figure 9:**
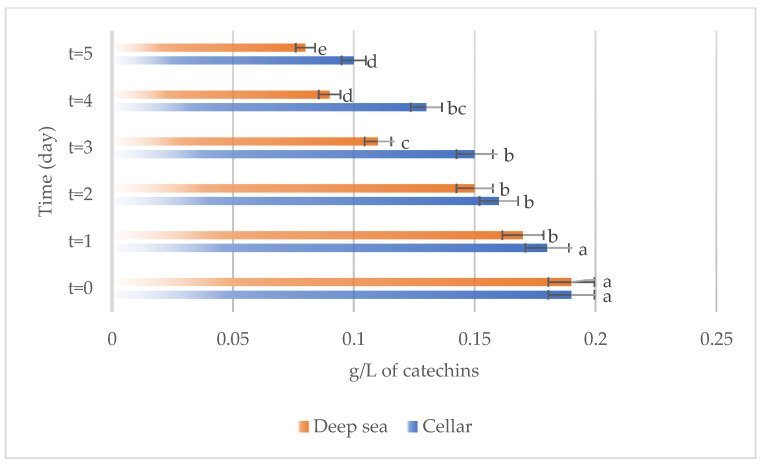
No flavonoids present on the samples considered (Sangiovese both under the sea and in the cellar). Letters (a–e) indicate significant differences (*p* < 0.05) over time, after ANOVA analysis of variance.

**Figure 10:**
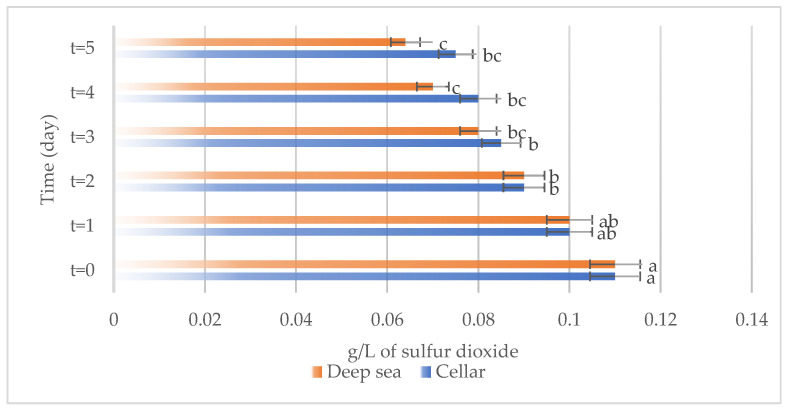
Total sulfur present on the samples considered (Sangiovese both under the sea and in the cellar). Letters (a–c) indicate significant differences (*p* < 0.05) over time, after ANOVA analysis of variance.

The development of different chemical compounds, including total phenols, total anthocyanins, non-flavonoid compounds, and total sulfur in Sangiovese wines stored under the two different conditions, specifically in the cellar or in the deep sea, over a period are reported in Figure 7, Figure 8, Figure 9 and Figure 10 respectively.

As demonstrated by the results, certain modifications in the chemical composition of Sangiovese wines occurred during the storage period. At time zero, the concentration of total phenols in the wines stored in the cellar and under the sea (Figure 7) were similar. However, over time, they underwent a gradual decrease in both storage conditions. This reduction may be related to chemical transformations or reactions occurring in the wine during the aging process.

Similarly, the levels of total anthocyanins (Figure 8), responsible for the wine color, exhibit a decreasing trend over time in the wines stored in both the investigated conditions, suggesting possible degradation or interaction of anthocyanins with other compounds present in the wine.

Non-flavonoid compounds (Figure 9), which encompass various chemical constituents of the wine, also showed a gradual decrease over time in both storage conditions, that could be attributed to chemical reactions, oxidation, or other processes occurring during the aging period [22].

Total sulfur dioxide (Figure 10) underwent a consistent decrease over time in the wines stored in both the cellar and the deep-sea conditions. This reduction in total sulfur levels may result from chemical reactions or volatilization processes taking place during storage.

The observed modifications in the compounds of Sangiovese wine highlighted the complex nature of wine aging and the influence of storage conditions. The changes observed in the chemical composition may impact the sensory characteristics and quality of the wine. Further research is necessary to elucidate the specific mechanisms and underlying factors contributing to these compound modifications.

This work analyzed the multifaceted world of wine aging, exploring how factors such as oxygen, temperature, storage conditions, and closure systems influence the composition of wine. Indeed, advanced analytical techniques were employed to meticulously monitor physical parameters and assess compositional changes, giving valuable insights into the mechanisms involved in wine aging. An intriguing aspect is the choice to compare two types of bottle closures, namely the classic cork and the crown or screw cap. Although both have similar oxygen permeability, the preference for cork reflects its widespread use and acceptance in the wine industry. This raises questions about traditions and conventions in the wine sector and the potential implications of changes in bottle closure approaches. Moreover, the thermodynamic analysis sheds light on the dynamic relationship between temperature and pressure within the wine bottles. The observed fluctuations in pressure, even with consistent temperature, hint at complex chemical reactions occurring within the wine matrix, likely influenced by oxygen interactions and other environmental variables. This aspect underscores the necessity for a comprehensive understanding of the physical and chemical dynamics involved in wine aging. Notably, preliminary findings suggested that aging wine underwater did not induce significant alterations in its fundamental characteristics compared to traditional cellar aging. This highlights the robustness of underwater aging as a potential alternative to conventional methods. Chemical analysis, in fact, showed slight but significant reductions in total phenols, anthocyanins, non-flavonoid compounds according to Monagas et al. [23], and sulfur dioxide in both Merlot and Sangiovese wines, regardless of storage conditions as observed in the literature [24]. These findings underscore the complexity of wine aging and the importance of storage conditions on its chemical composition, crucial for maintaining quality and achieving desired sensory characteristics.

## 4. Conclusions

The present research was conducted to investigate the feasibility of aging high-quality red wines under the sea. The methodologies employed in this study encompassed a range of sophisticated analytical tools, including but not limited to high-resolution altimeter sensors, laser spectroscopy-based technologies for atmospheric control, and standard chemical analysis methods. These methodologies were meticulously applied to gather comprehensive data on the physical and chemical aspects of wine aging, enabling a thorough examination of the involved processes. Traditional cellar storage ensures a stable temperature and minimal exposure to light, ideal for gradual maturation. However, the emergence of underwater ageing represents a new approach, inspired by historical discoveries of well-preserved wines from sea wrecks. Investigating how this unconventional method influences the chemistry profiles of wine could lead to innovative practices in the industry. The chemical analyses carried out in the study offered a comprehensive view of the changes that occur in the composition of wine during ageing. Preliminary findings from this investigation indicate that the process of aging wines under the sea does not induce significant alterations in their fundamental characteristics. Indeed, both storage methods induced changes in wine composition, with variations observed in phenolic compounds, anthocyanins, and sulfur dioxide levels. These results have implications for the understanding of fermentation processes, particularly in the context of ancestral wines, as well as the bottle re-fermentation method commonly employed in classic winemaking. It is worth noting that these findings extend beyond the wine industry and have potential applications in other domains, such as the production of oil, where exposure to oxygen can result in detrimental effects on product quality. While the preliminary results of this study are promising, further investigations are necessary to comprehensively elucidate the intricacies of sea aging and to explore the broader implications of this research. Overall, this study could bring valuable insights into the complex synergy of factors influencing wine aging, paving the way for future research and potential advancements in winemaking practices.

## Figures and Tables

**Table 1:** All samples that were used for experimentation.

Wine Type	Cork Type	Capsule Type	Wrapping	Bottle Orientation	Storage Location
Sangiovese 2019	Monoblock cork	Shellac capsule	Wrapped	Held upright	Under sea
Merlot 2020	Monoblock cork	Shellac capsule	Wrapped	Held upright	Under sea
Sangiovese 2019	Monoblock cork	Shellac capsule	Wrapped	Held upright	Cellar
Merlot 2020	Monoblock cork	Shellac capsule	Wrapped	Held upright	Cellar

**Table 2:** Chemical composition at the beginning of experimentation for Merlot and Sangiovese.

Parameters	Merlot	Sangiovese
Ethanol (% vol/vol)	15.02 ± 0.01 ^b^	14.03 ± 0.01 ^a^
Sugar (g/L)	3.06 ± 0.03 ^b^	3.13 ± 0.01 ^a^
Hexoses (g/L)	1.21 ± 0.01 ^a^	0.93 ± 0.03 ^b^
pH	3.47 ± 0.01 ^a^	3.35 ± 0.01 ^b^
Titratable acidity (g/L)	5.63 ± 0.01 ^b^	6.1 ± 0.01 ^a^
Volatile acidity (g/L)	0.76 ± 0.01 ^a^	0.53 ± 0.01 ^b^
Malic acid (g/L)	0.010 ± 0.01 ^b^	0.69 ± 0.02 ^a^
Lactic acid (g/L)	0.89 ± 0.01 ^a^	0.37± 0.03 ^b^
Tartaric acid (g/L)	2.54 ± 0.01 ^b^	2.79 ± 0.02 ^a^
Citric acid (g/L)	0.030 ± 0.01 ^b^	0.15 ± 0.01 ^a^
Total dry matter (g/L)	33.05 ± 0.10 ^a^	31.46 ± 0.03 ^b^
Glycerin (g/L)	10.07± 0.02 ^a^	8.95 ± 0.01 ^b^
Potassium (g/L)	0.89 ± 0.01 ^a^	0.85 ± 0.01 ^b^

The letters highlighted significant differences between the wines assessed by one-way ANOVA (*p* < 0.05).

## Data Availability

The original contributions presented in the study are included in the article, further inquiries can be directed to the corresponding author.
